# Mechanosensing of Stimuli Changes with Magnetically Gated Adaptive Sensitivity

**DOI:** 10.1021/acsmaterialslett.4c02021

**Published:** 2025-02-04

**Authors:** Xichen Hu, Xianhu Liu, Quan Xu, Olli Ikkala, Bo Peng

**Affiliations:** † Department of Applied Physics, Aalto University, P.O. Box 15100, FI 02150 Espoo, Finland; ‡ Center of Excellence in Life-Inspired Hybrid Materials (LIBER), Aalto University, P.O. Box 16100, 00076 Aalto, Finland; § State Key Laboratory of Heavy Oil Processing, China University of Petroleum (Beijing), Beijing 102249, China; ⊥ Department of Materials Science and Engineering Research Center for Advanced Coatings of Ministry of Education, Fudan University, Shanghai 200433, China

## Abstract

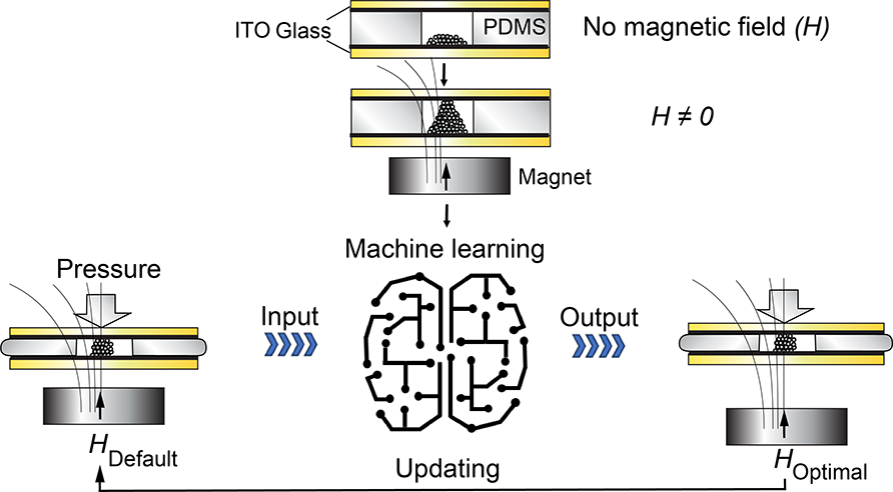

Inspired by biological sensors that characteristically adapt to varying stimulus ranges, efficiently detecting stimulus changes sooner than the absolute stimulus values, we propose a mechanosensing concept in which the resolution can be adapted by magnetic field (*H*) gating to detect small pressure-changes under a wide range of compressive stimuli. This is realized with resistive sensing by pillared *H*-driven assemblies of soft ferromagnetic electrically conducting particles between planar electrodes under a voltage bias. By modulation of *H*, the pillars respond with mechanically adaptable sensitivity. Higher *H* enhances current resolution, while it increases scatter among repeating measurements due to increased magnetic structural jamming between colloids in their assembly. To manage the trade-off between electrical resolution and scatter, machine learning is introduced for searching optimum *H* gatings, thus facilitating efficient pressure prediction. This approach suggests bioinspired pathways for developing adaptive stimulus-responsive mechanosensors, detecting subtle changes across varying stimuli levels with enhanced effectiveness through machine learning.

Remarkable advances have been made over recent years in stimulus-responsive materials.^1−4^ A wealth of stimuli, including pH, temperature, humidity, light, electric field, and magnetic field, have been used to drive different responses, also suggesting stimulus-responsive materials for sensors.^5−12^ Among sensors, mechanosensors are increasingly relevant, upon converting the applied mechanical or haptic stimuli into electrical or other signals, based on resistance, capacitance, or voltage.^13−16^ Resistive pressure sensors show advantages in simplicity in their structures, ease of the signal acquisition, and high sensitivity.^15,17−21^ On the other hand, colloidal particles have attracted growing attention in sensing.^22−27^ For instance, magnetic patchy colloidal particles with conductive coatings suggest pressure sensors with remarkable sensitivity, involving magnetic and electrical functionalities.^27^

Classically, sensors in engineering are designed to detect absolute signal values within their predefined stimuli ranges aiming at fixed, stable, and high sensitivity. By contrast, biological systems characteristically sense signal changes adaptively under different conditions, e.g., to warn of threats or to allow homing under Earth’s magnetic field.^28−32^ They inspire an exploration of avenues for emerging autonomous soft robots, in particular to detect environmental changes to control their behavior.^33−37^ Among the biological sensing architectures, hairlike structures are ubiquitous, ranging, e.g., from cochlear hearing by animals to mechanosensing of hydrodynamic flows by fish, caterpillars, scorpions, and bats.^38−40^ They suggest exploring hair-like or pillared surface topographies for adaptive bioinspired mechanosensings. More generally, adaptive mechanosensing remains a great challenge using synthetic materials.

Herein, we show mechanoresponsive sensing concepts that allow a rudimentary dynamic adaptation to efficiently resolve small signal changes with small scattering under different stimulus ranges. This is facilitated by magnetic field (*H*) driven electrically conducting pillared assemblies of magnetic colloidal particles between electrodes, where the complex response is further rationalized by machine learning. As the working logic (Figure 1), electrically conducting ferromagnetic cobalt colloids (ECFCCs) assemble to tunable hair-like pillars between two planar electrodes driven by the strength of the exposed *H*, which is used as a gating signal. Upon exposure to pressure stimuli, the pillars undergo structural deformations, thus leading to changes in the electrical resistance across the pillars, thereby enabling mechanoresponsivity.

**Figure 1:**
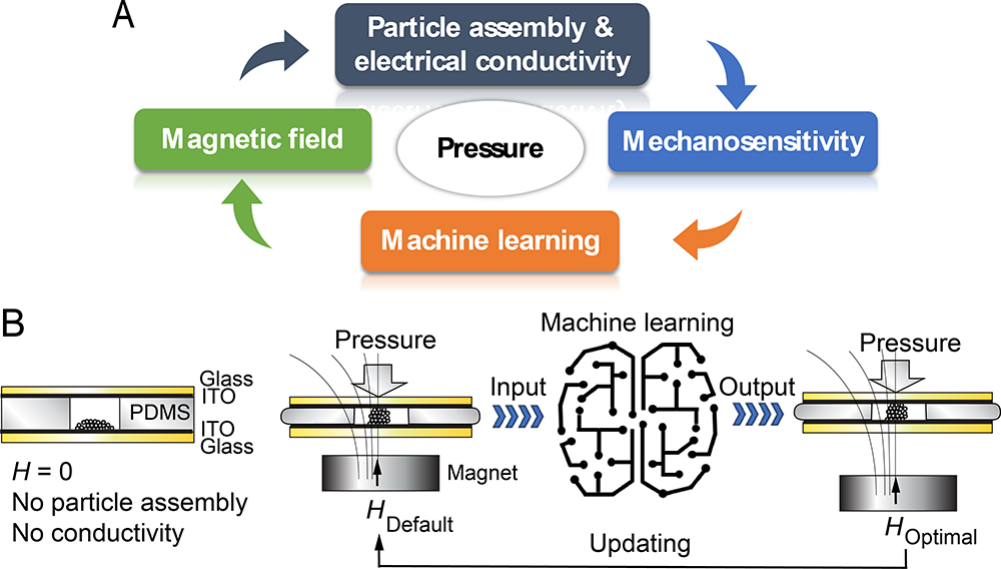
Logic (A) and workflow (B). Upon exerting a magnetic field, the soft ferromagnetic colloidal particles assemble as electrically conducting pillars between two planar electrodes to form a resistive mechanosensor. The pillar assemblies respond adaptively to compression, as gated by different magnetic fields, aiming to resolve compressive stimuli changes.

The ECFCCs are synthesized based on a modification of our previous protocols^22,41,42^ using CoCl_2_·6H_2_O, trisodium citrate, and NaOH in diethylene glycol at 210 °C, leading to chemical reduction to citrate-stabilized metallic cobalt (Co). Scanning electron microscopy shows colloidal Co particles with an average diameter of 5.32 ± 0.33 μm consisting of nanometric subdomains, leading to surface irregularities (Figure 2A–C). The presence of Co is confirmed by energy dispersive X-ray spectroscopy (Figure 2D). At 300 K, the ECFCCs are softly ferromagnetic, with a small coercivity and a remanence of ∼213.4 Oe and ∼9.1 emu/g, respectively (Figure 2E). As discussed later, they allow resistive mechanosensors upon the formation of *H*-driven electrically conducting pillared assemblies where the conductivity value is gated by the *H* value (Figure 2F).

**Figure 2:**
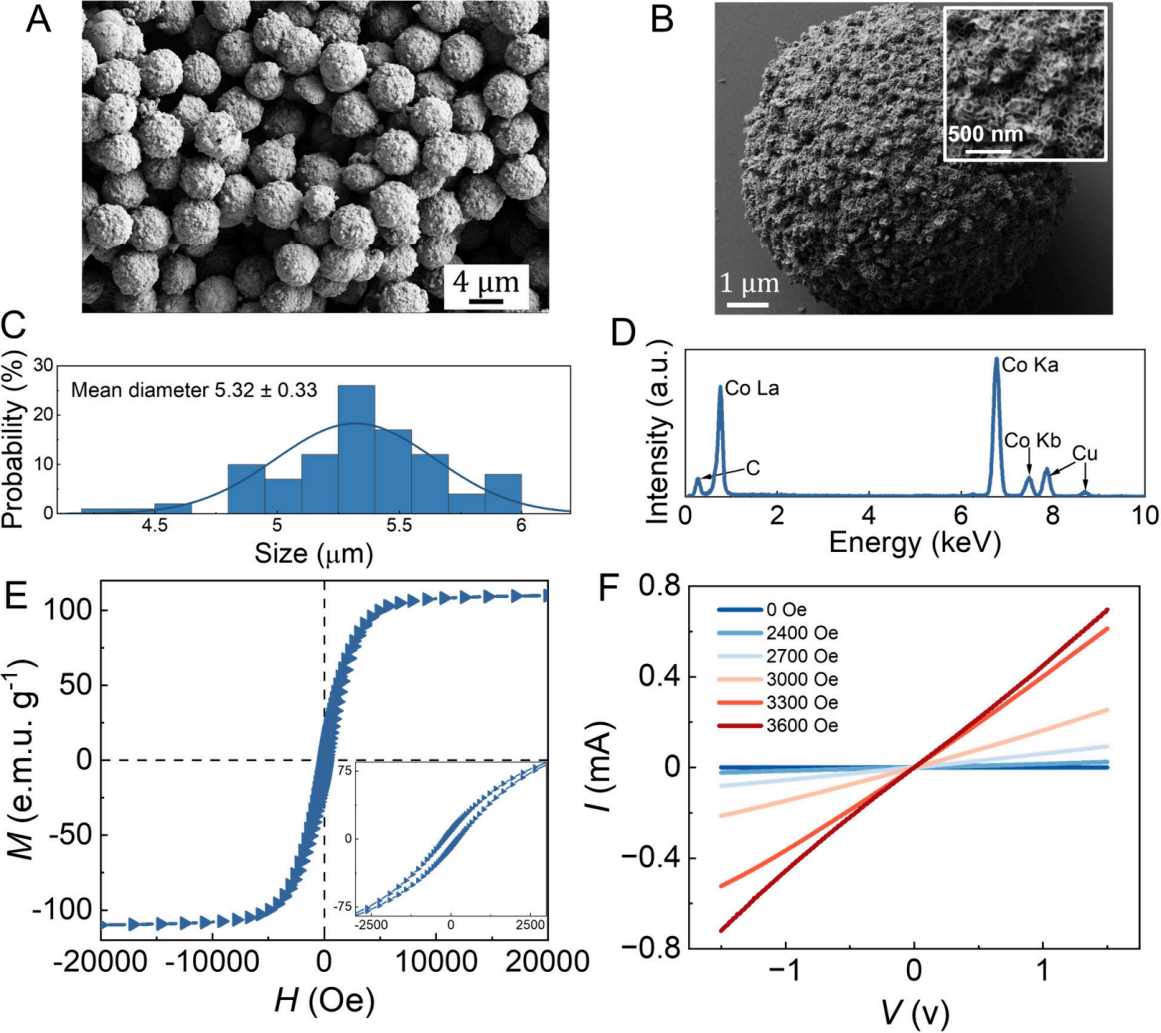
A scanning electron micrograph of (A) cobalt colloidal particles and (B) a high resolution micrograph of one cobalt particle. (C) Size distribution thereof. (D) Energy dispersive X-ray spectroscopy confirming the chemical nature of Co particles. (E) Mass magnetic moment *M* as a function of the exposed magnetic field *H* at 300 K. (F) Linear sweep voltammetry indicating the *H*-dependent conductivity of the Co-particle-based *H*-driven pillars beyond a threshold magnetic field. The conductivity is tunable upon gating by *H*. Voltage sweep is applied from −1.5 to 1.5 V with an electrode spacing of *d* = 1.2 mm.

Soft ferromagnetic colloids assemble into electrically conducting hair-like pillars upon exposed magnetic fields.^22^ To exploit this for adaptive mechanosensing, here we investigate the effect of pressure as a mechanical stimulus on the electrical resistance between two planar electrodes after the pillars have been initially formed at different *H. H* is applied at the site of ECFCCs by a permanent neodymium magnet positioned at a tunable distance *z* below the lower electrode, where the field strength and gradients are shown in Figure S1. Controlled amounts of ECFCCs are confined between two indium tin oxide (ITO) coated glass electrode plates (scheme in Figure 3A and compositional details in SI). The initial electrode separation *d* is controlled by a soft polydimethylsiloxane (PDMS) elastomer layer between the electrodes, which involves a punched cavity with an area of 15 × 15 mm^2^ to confine a defined amount of ECFCCs (see SI for compositional details). The elasticity of the PDMS layer allows mechanical compression toward reduced *d* (Figure 3A, Figure S2A). In the absence of *H*, ECFCCs are evenly distributed without pillars on the ITO glass slide, with no conduction between the electrodes. When *H* is applied, they assemble into truncated cylinder-like micropillars due to magnetic dipole–dipole interactions along the *H* direction (see ref (22) for a generic shape discussion). When the electrical conductivity of ECFCCs and the magnetic field strength required to form the pillars are taken into account, an electrical continuity is established between two ITO-coated glass electrodes, thereby allowing a current *I* to flow upon exposing a bias voltage *V* (Figure 2F).

**Figure 3:**
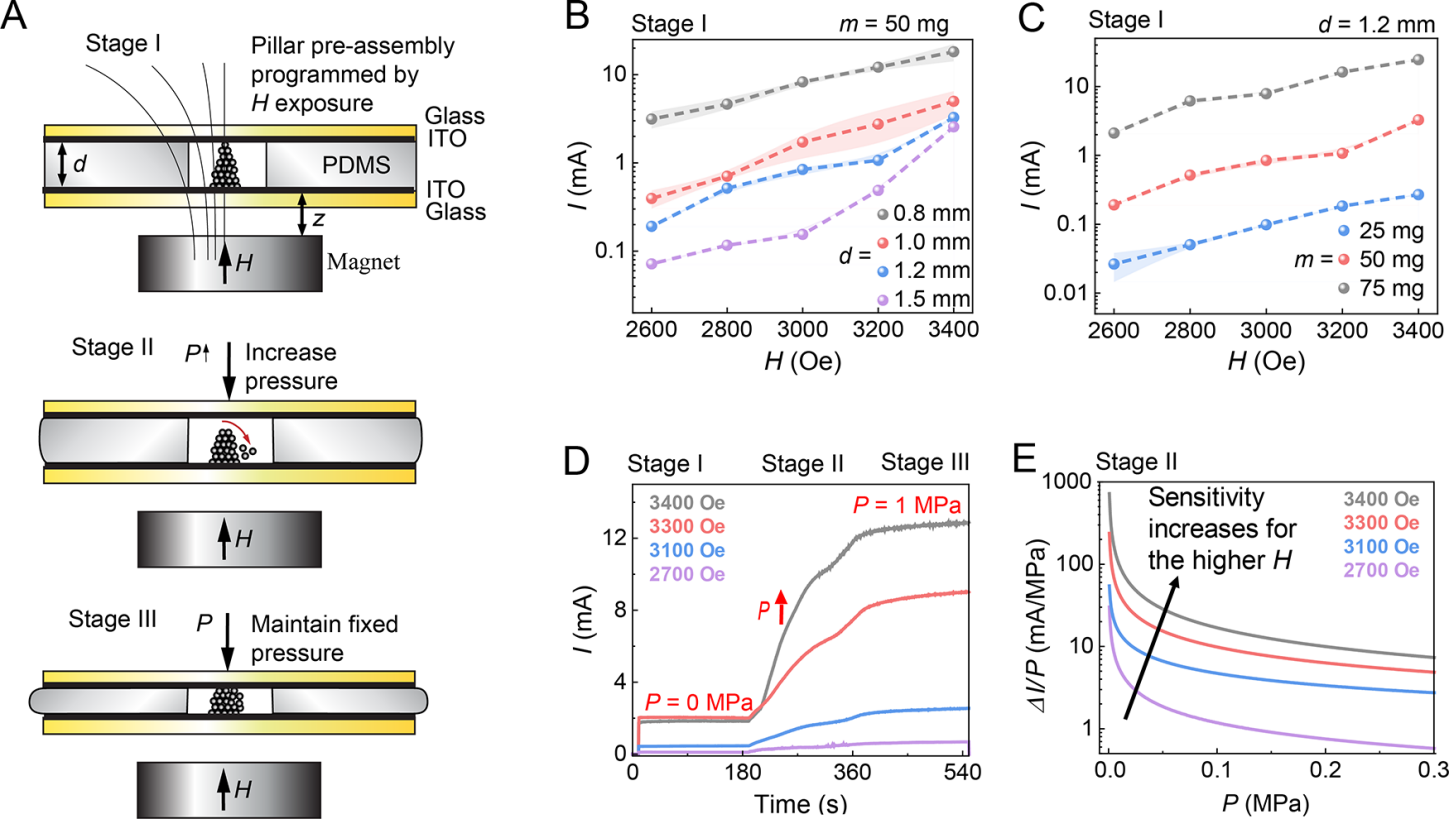
(A) Schematics of a magnetic-field-gated mechanosensor to detect pressure changes. The electrical response involves three stages: (Stage I) First, ECFCCs are initially assembled into electrically conducting pillars using an exposed magnetic field *H* by controlling the distance *z* between the magnet and the system. (Stage II) Gradual increase of the pressure *P* (from zero to for example 1 MPa). (Stage III) Maintaining the fixed pressure until the pillars and the resulting electrical current reach an equilibrium. (B) The equilibrium currents *I* as a function of *H* at different separations *d* between the two electrodes before compressing (stage I). The sample mass is *m* = 50 mg under an area of 15 × 15 mm^2^. (C) Resulting *I* as a function of *H* across different sample loadings. The initial separation between two electrodes is *d* = 1.2 mm (stage I). (D) Compressive response of *I* corresponding to the three stages depicted in Figure 3A under different *H* values for the initial *d* = 1.2 mm. The extent of PDMS compression is predefined and monitored using an Instron machine, with a set compression rate of 10% of *d* per minute up to a maximum strain of 20%. (E) The rate of scaled current variation Δ*I*/*P* as a function of pressure *P* (mA/MPa; applied over a total area of 75 × 25 mm^2^) in stage II, under different *H*, reveals the tunability of the response sensitivity of the mechanosensor. Data are given as mean ± SD (*n* = 3).

The softness of the PDMS layer allows for reduced *d* by mechanical compression (Figure 3A) after the pillars have been formed at different *H*. To investigate the effects thereof, the initial separation is first set as *d* = 1.2 mm. *H* is applied on ECFCCs (50 mg in the area 15 × 15 mm^2^, see compositional details in SI), leading to their assembly into pillars and electrical conduction (Figure 3B, denoted as stage I). Higher conductivity is observed upon increasing the exposed magnetic field due to pronounced pillar formation or reducing the initial *d* (Figure 3B and Figures S3–S5). This is explained by a simple model assuming truncated cylindrical pillar shapes, wherein the conductivity is limited by the upper truncated surface area against the upper electrode.^22^ Not surprisingly, when the amount of ECFCC in the specified electrode area is increased (Figure S6), the conductivity is increased, and the conductivity increases roughly exponentially as a function of *H* (Figure 3C and Figure S6). Such an initial uncompressed state is depicted in Figure 3D (stage I, during the first 180 s). Next, compression is applied using an Instron machine (Figure 3D), denoted as stage II. This leads to a gradually reduced *d* and reassembly of pillars. At the end of compression, *d* reduces to 0.96 mm from 1.2 mm (i.e., 20% reduction of *d*) and remains constant, whereupon *I* stabilizes to a steady-state value (Figure 3D). The final pressure was set by the Instron machine to a constant value of 1 MPa; see Figure 3D. The current values were repeatable upon cycled compressions and decompressions (Figure S7). The sensitivity during stage II can be quantified by recording the current change (Δ*I*) from the uncompressed initial state and scaling it with the instantaneous applied pressure *P* during continuously increasing compression (Figure 3E). The sensitivity is promoted during the early stages of compression upon increased *H*.

Next, we explore the magnetically gated adaptive mechanosensing upon applying a wide range of final constant pressures using a fixed pressure at *d* = 1.2 mm. First, *H* = 3100 Oe is applied to initially assemble ECFCCs into pillars, whereupon the corresponding *I* is recorded and presented as the red line in Figure 4A. Subsequently, tunable pressures based on a water filled beaker were positioned over the total glass-electrode area of 75 × 25 mm^2^. Thus, a constant pressure *P* = 1.05 kPa is positioned on the top center of the ECFCC sensor, leading to the compression of *d* (Figure 4B). As a result, the measured *I* increases due to the pillar reassembly and a new *I* steady-state value is reconstituted in seconds (red line in Figure 4B). Some variations are expected due to the different placement of such a weight vs the sensor area. To quantify the effect, the weight was placed at eight different locations, and the corresponding changes in the responsive current are recorded and presented as the red lines in Figure 4C. These variations in electrical signals, caused by positional differences, are considered as data scatter and are incorporated into the subsequent modeling to account for this variability.

**Figure 4:**
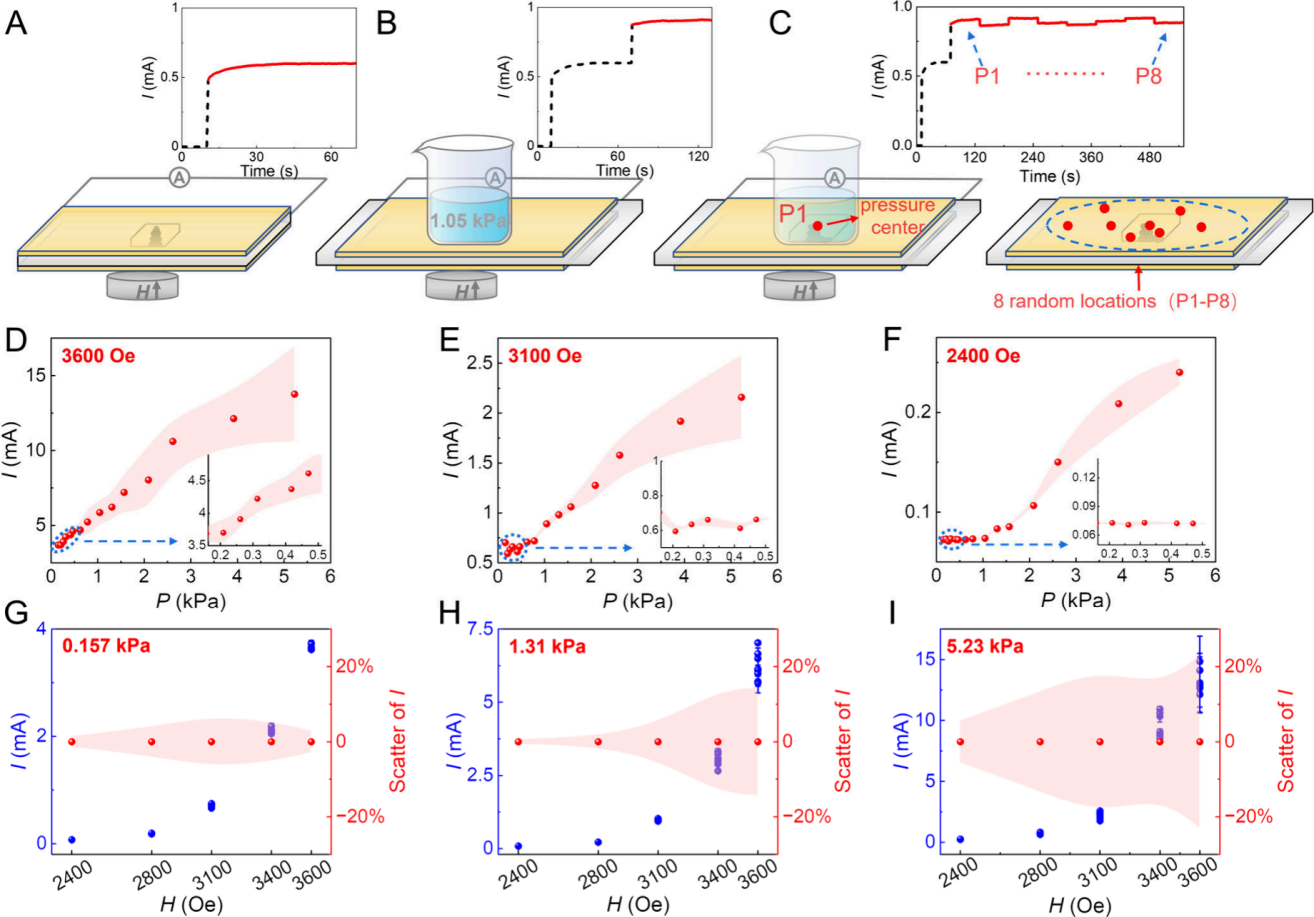
(A–C) Schematics for the data collection where the pressure sensitivity is demonstrated by weighing different weights as compressive pressures. (D–F) Correlation between the exposed pressure (*P*) and the response current (*I*) during the weighing process under *H* = 3600 Oe (D), 3100 Oe (E), and 2400 Oe (F; see also Figures S9–S11). The insets show the small pressure ranges. (G–I) The response currents *I* (blue points) and their normalized scatter (red data points and scatter, as indicated by red shading) resulted from applied pressures of 0.157 kPa (G), 1.31 kPa (H), and 5.23 kPa (I) during five magnetic field exposures. Data are given as mean ± SD (*n* = 3).

Next, the sensitivity to resolving pressure changes is explored upon different *H* gatings. First, the reproducibility is explored by applying pressures ranging from 0.157 to 5.23 kPa at different locations using three *H* gatings. The corresponding *I* values are recorded (Figure S8), where the average *I* values at eight different locations and different *H* gatings are depicted in Figures S9–S11. As summarized in Figures 4D–F, the current resolution to efficiently resolve small pressure changes is tunable for different pressure ranges by using various *H* gatings above the threshold value 2400 Oe. A high resolution to detect small pressure changes for low pressures (<ca. 0.5 kPa) is achieved by incorporating large magnetic fields, such as *H* = 3600 Oe and *H* = 3100 Oe (Figures 4D,E). By contrast, to resolve small pressure changes for high pressures suggests using low magnetic fields, such as *H* = 2400 Oe (Figure 4F).

However, the scatter between consecutive measurements is higher using a larger *H*. Therefore, the resolution and scatter are competing. This is probably due to the stronger structural jamming effects in the pillar assemblies/disassemblies caused by higher magnetic dipolar interactions at higher *H*; the magnetic jamming between soft ferromagnetic colloids is caused by the combination of surface roughness and permanent magnetic dipolar–dipolar interaction of the colloids.^22,43,44^ Soft ferromagnetic particle jamming in *H*-driven assemblies was explicitly explored recently.^22,42^ Thus, the resolved *I* values under a strong field will significantly suffer from pronounced scatter in successive measurements (Figures 4G–I). This suggests that a weaker field should be given priority therein. For the pressure of 0.157 kPa in Figure 4G, the measurements gave a small scatter at both the lowest and the highest fields. But, due to the reduced resolution (Figure 4F), therein, the low field (i.e., 2400 Oe) is not optimal. In summary, achieving high-resolution of pressure changes favors a large *H*, as it produces a steep current vs magnetic field slope. However, to minimize scatter, a smaller magnetic field—still above the sensing threshold for pillar formation—is preferred. This introduces complexity into the system, which is further addressed next.

For a deeper understanding of the results and selecting the optimal *H* gating, thus enabling reliable mechanosensing at different pressure ranges, we incorporate machine learning (ML)^45,46^ as illustrated in Figure 5A. The ML model serves two purposes, i.e., optimal *H*-gating selection and pressure prediction. For an unknown pressure, a default magnetic field (*H*_default_) is first applied to generate a stable current *I* for an initial estimation (*P*_e_) of the unknown pressure. Then, we estimate the optimal gating *H*_opt_ for the target pressure through polynomial ML models (Figure S12). A new stable current *I*′ generated under *H*_opt_ will be applied to better predict the actual predicted pressure *P*_pre_.

**Figure 5:**
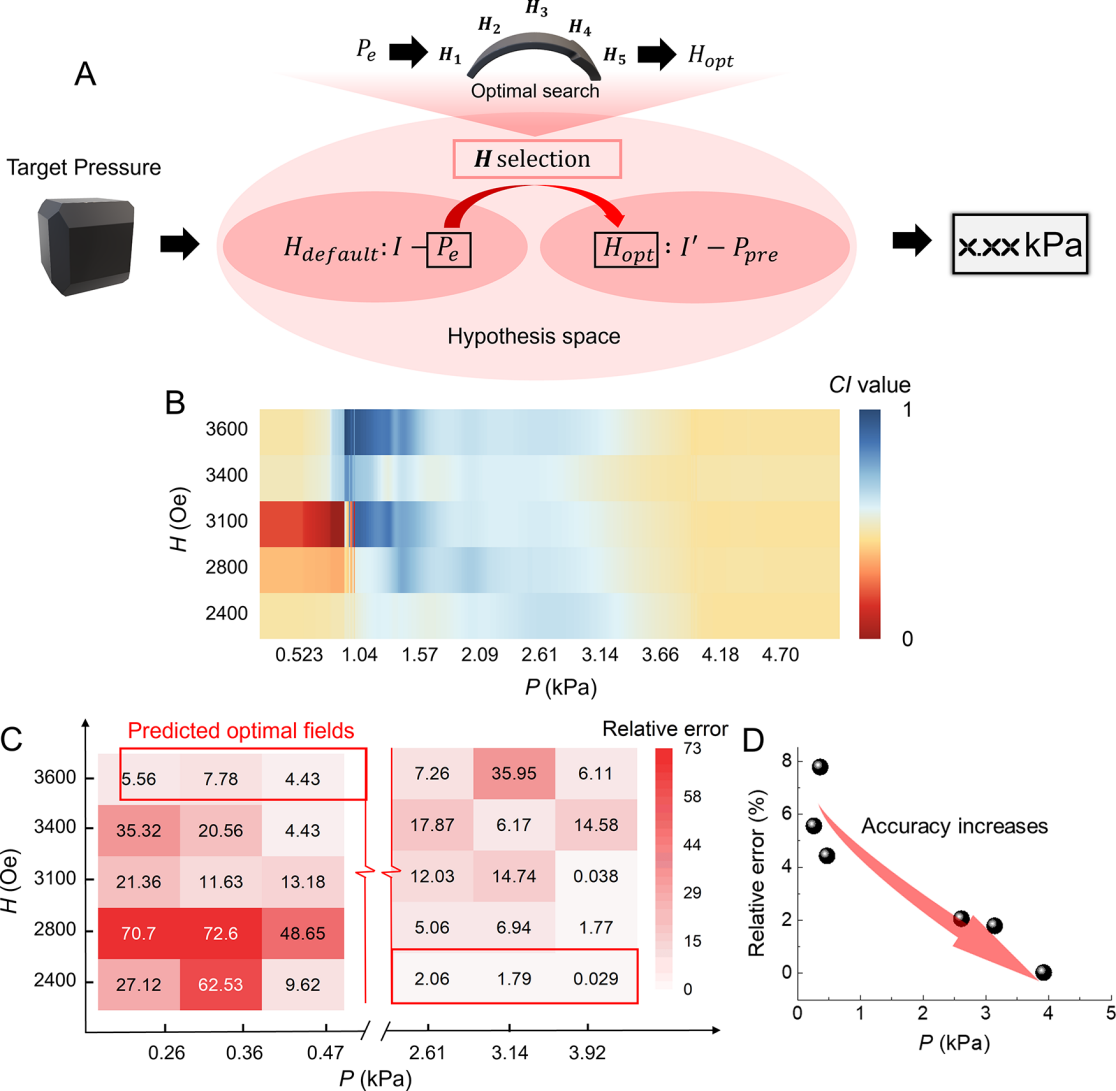
(A) Schematics illustrating the machine-learning-based analysis and compressional mechanical pressure prediction. *H*_default_ is the initial *H* applied to estimate the exerted pressure (*P*_e_) by measuring the current (*I*). *H*_opt_ is the optimal field for pressure prediction, where *I*′ denotes the current as remeasured under *H*_opt_. *P*_pre_ is the final predicted pressure. The selection of *H* involves an optimal search using *P*_e_ as the input upon fitting for the current–pressure pairings. (B) Continuous distribution of the coupling index (*CI*) values used for the *H* selection; the optimal field exhibits the highest *CI* value within the corresponding pressure range. *CI* is a metric that quantifies field reliability (Figure S13). (C) Predicted results from the optimized mechanosensing system represented by relative error (absolute error divided by the true value). Red boxes highlight the optimal fields selected by the model. (D) Trend of the prediction error as the pressure increases.

Therein, we use the Gaussian process regression (GPR) algorithm^47,48^ to capture the data correlations, wherein we define the interplay of “local slope” and “local scatter” for resolution and data scatter (Figures S13 and S14). Then, a coupling index *CI* = local slope/local scatter is introduced as a metric, where the magnetic field with the highest *CI* value is deemed as optimal under different pressure ranges. Figure 5B summarizes the distribution of *CI* values across all conditions, calculated from our data (Figure S15). It suggests that, as the pressure increases, the optimal *H* decreases, consistent with the trend observed in Figure 4D–I.

To demonstrate the quantitative effectiveness of the ML for the mechanosensing system, we tested six additional pressures and the predictions as illustrated in Figure 5C. They are converted to relative errors with respect to the actual values. First, the system successfully predicts the optimal fields (highlighted in red box) for pressures at different levels, as validated by the minimal relative errors. The relative errors are found to increase as the pressure rises (Figure 5D), likely due to the enhanced sensitivity of conducting pillars to external pressure. The results demonstrate the capability of the designed mechanosensory system with an effective ML analysis.

In summary, we show a concept for adaptive bioinspired mechanosensing to detect small pressure changes, combining small scatter under widely different pressure ranges by recording the electrical responses of electrically conducting, magnetically driven assemblies of soft ferromagnetic magnetic particles supported by machine learning. Therein, we show adaptive mechanosensing with tunable mechanosensitivity based on the external magnetic field gating on the particle assembly to efficiently resolve compressional mechanical changes. To allow predictive power, we developed a machine learning-guided computational network that enables optimization of the sensitivity vs scatter to detect small pressure changes where the range is tunable by exposed magnetic field. This work introduces a paradigm toward the design of bioinspired sensors for dynamically adaptive mechanosensors to detect stimuli changes, paving ways to engineer, e.g., soft robot behaviors.

## Supporting Information Available

Materials, preparation, and characterization of ECFCCs; machine learning method of data analysis; schematics of measurement setup; magnetic field profile (Figure S1); setup (Figure S2); pillar assembly schematics (Figure S3); current-dependent magnetic field and pressure (Figures S4–S6); repeatability test (Figure S7); scatter (Figure S8); *H*-dependent data under variable pressure (Figure S9) and their analysis (Figure S10); the correlation between *P* and *I* (Figure S11); polynomial fitting of data (Figure S12); the workflow of optimizing the *H* (Figure S13); GPR fitting of data (Figure S14); prediction of optimal *H* (Figure S15); model evaluation (Table S1); code for the pressure prediction (Note S1) (PDF)
