# Supplementary material for: Mechanosensing of Stimuli Changes with Magnetically Gated Adaptive Sensitivity
Source: ACS Mater Lett. 2025 Feb 4;7(3):862–8. doi: 10.1021/acsmaterialslett.4c02021 (PMC11881142; doi:10.1021/acsmaterialslett.4c02021)
Supplement: Supplementary file 1 — tz4c02021_si_001.pdf [file tz4c02021_si_001.pdf]

Supporting Information

Mechanosensing of Stimuli Changes with Magnetically Gated Adaptive Sensitivity

Xichen Hu[1,2,+], Xianhu Liu[1,2,+], Quan Xu[3], Olli Ikkala[1,*] and Bo Peng[1,2,*]

[1] Department of Applied Physics, Aalto University, P.O. Box 15100, FI 02150, Espoo, Finland

[2] Department of Materials Science and Engineering Research Center for Advanced Coatings of Ministry of Education, Fudan University, Shanghai 200433, China

[3] State Key Laboratory of Heavy Oil Processing, China University of Petroleum (Beijing), Beijing, 102249, China

[+] Equal contribution

Experimental Section

Materials

Diethylene glycol (DEG, ≥99.5%) was purchased from Fisher Scientific. Ethanol (≥99.5%) was provided by Altia Oyj, Finland. Sodium citrate tribasic dihydrate (NaCit, ≥99.0%), cobalt(II) chloride hexahydrate ($CoCl_2$ $6H_2O$, 98.0%), and sodium hydroxide (NaOH, ≥98.0%) were obtained from Sigma-Aldrich. Deionized water from a Millipore Direct-Q UV 3 reverse osmosis filter apparatus was used in all experiments. All chemicals were used as received.

Preparation of ECFCCs

The synthesis is based on a high temperature polyol method.[1-3] Typically, 0.02 mol NaOH was dissolved in 15 ml of DEG by magnetic stirring at 150 °C. Meanwhile, in a 250 ml flask equipped with a N_2 inlet, a condenser, and mechanical stir shaft, a mixture comprising of 0.008 mol $CoCl_2.6H_2O$ and 0.0054 mol NaCit and 15 ml DEG was heated up to 210 °C in a N_2 atmosphere and maintained for 30 min while mechanically stirring at 220 rpm. Then, the as-prepared NaOH/DEG mixture was rapidly injected into the $CoCl_2$/NaCit/DEG phase. After 5 hours, the reaction was cooled to room temperature naturally, and Co particles were magnetically decanted. Prior to use, the Co particles were rinsed three times with ethanol and water, then dried in vacuum at room temperature, and finally stored in a glove box.

Compositional details

For the setup of the mechano-sensor as shown in Figure 3A, the default compositional details are particle mass m = 50 mg, electrode spacing (also the thickness of PDMS) d = 1.2 mm, and cavity volume v = $15 \times 15 \times 1.2$ mm^3, confining the particles. A permanent neodymium magnet is positioned beneath the particles, enabling control of the H strength by adjusting its vertical position. The H strength was measured at the bottom surface of the lower slide, coaxial with the magnet. Compressional pressure was applied over an area of 75×25 mm^2, centrally located on top of the magnet.

Characterization

<u>Scanning electron microscopy</u>

A Zeiss Sigma VP scanning electron microscope (SEM) at an operating voltage of 1 kV was used to analyse the surface morphology and to get a global overview of the ECFCCSs.

<u>X-Ray diffraction</u>

The crystal structure analysis of the powder samples was conducted using X-ray diffraction (XRD) patterns recorded with a Rigaku SmartLab X-ray diffraction, operating at 35 kV and 15 mA with Cu Kα radiation (λ = 0.15406 nm).

<u>SQUID-magnetometry</u>

The magnetic properties were characterized using a magnetic properties measurement system (MPMS-XL7, Quantum Design), equipped with a superconducting quantum interference device (SQUID) magnetometer. Measurements of magnetic moment and susceptibility were

performed at a temperature of 300 K, with an applied magnetic field ranging from -20000 to 20000 Oe.

Machine learning (Note S1)

Data input

Pairs of data consisting of exposed pressure (*P*) and resulting current (*I*) are inputs, with the magnetic field (*H*) as their labels. Features (*P* & *I*) are processed with data standardization respectively. Sample size is 600 × 2.

Gaussian process regression

Gaussian process regression[4-5] (GPR): Gaussian process modelling is used on the regression fitting for external pressure *P* vs. current *I*, by leveraging from the confidence interval, that comprises a measure of uncertainty derived from the predictive distribution, and further being developed as one of the criteria (scatter) for the subsequent classification task – *H* selection (Figure S13). Then, by systematically iterating through the pressure range, all possible pressure values will be predicted and classified according to the predefined *H*-selection model (Figure S15). For the pressure prediction, polynomial models will be employed to predict the final pressure based on the given input *I* (Figure S12), measured under the optimal *H*.

H - selection

An input *I* measured at the default magnetic field H_{default} will be utilized to estimate the initial pressure P_{e} using GPR models. Then, we define the 'local scatter' and 'local slope' based on the GPR models (Figure S13) and calculate the coupling index (*CI*) as described in (Equation S1). The *CI* metric IS employed to determine the optimal field H_{opt}. In the *H*-selection module, the initial estimated pressure P_{e} serves as the input, and the optimal field mode H_{opt} is produced as the output.

Model evaluation

For regression models of GPR and polynomial fitting, mean squared error (MSE) and coefficient of determination (R^2) as the leading metrics are summarized in Figure S12 and Table S1.

Supplementary Figures and Tables

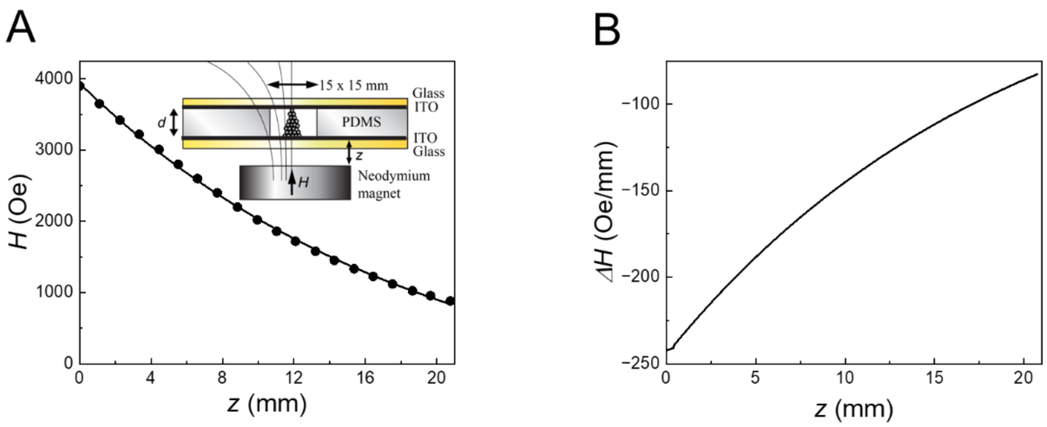

Figure S1. (A) Magnetic field strength on the magnet axis as a function of distance (z) from the surface centre of the magnet, showing the experimentally measured data (dots) and theoretical fits (lines). Inset, the measurement setup. (B) The corresponding vertical field gradients $\Delta H = dH/dz$.

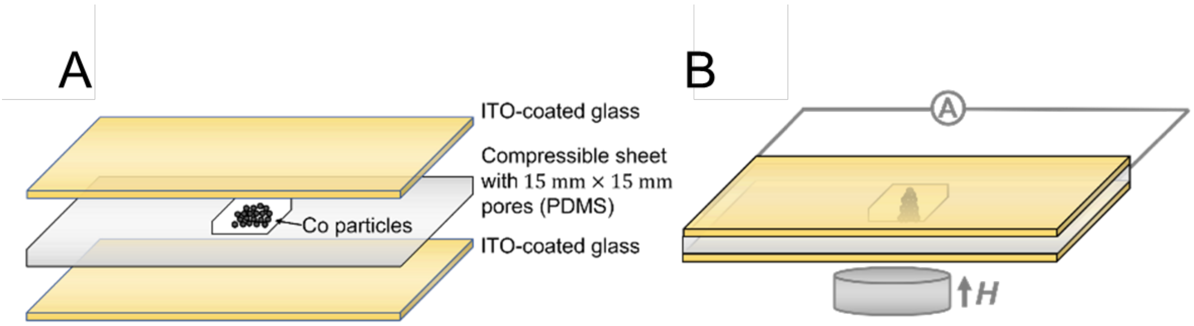

Figure S2. Schematics of the experimental setup used for investigating magnetic field gated electrical conductivity of ECFCCs for mechanosensing. (A) The sandwich structure. (B) The magnetic field gating.

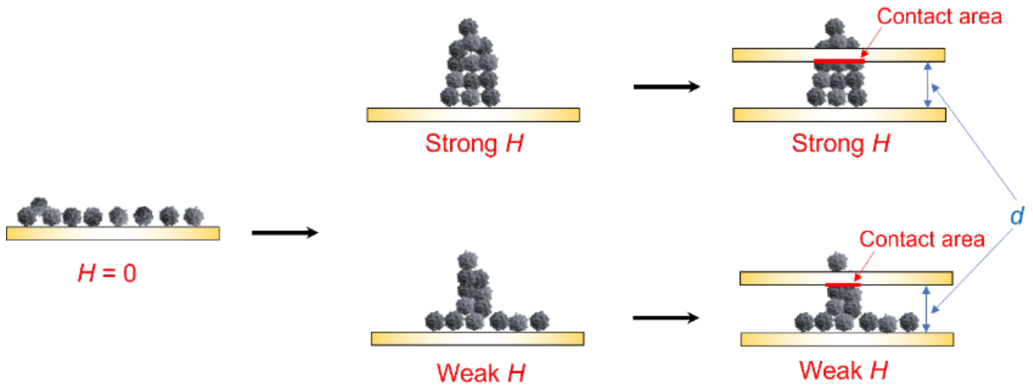

Figure S3. The pillar assembly is governed by the magnetic field *H*. Higher *H* leads to larger assembled pillars, resulting in an increased contact area with the upper electrode. The shape of the pillars can be approximated to be truncated cones, where the upper truncated area defines the electrical conduction.

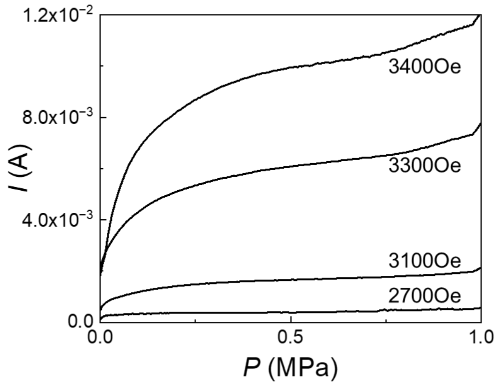

Figure S4. The graph depicting the relationship between electrical current and pressure variation at different exposed magnetic field gatings.

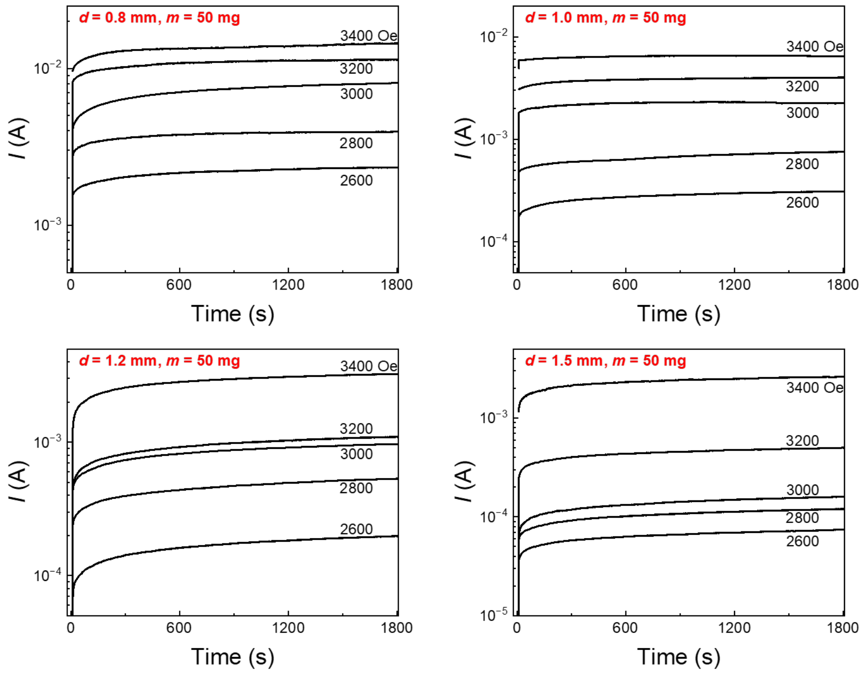

Figure S5. *d*-dependent electric current across ECFCCs under different magnetic field gating. The applied sample mass is m = 50 mg, the area of the ECFCCs is 15 × 15 mm.

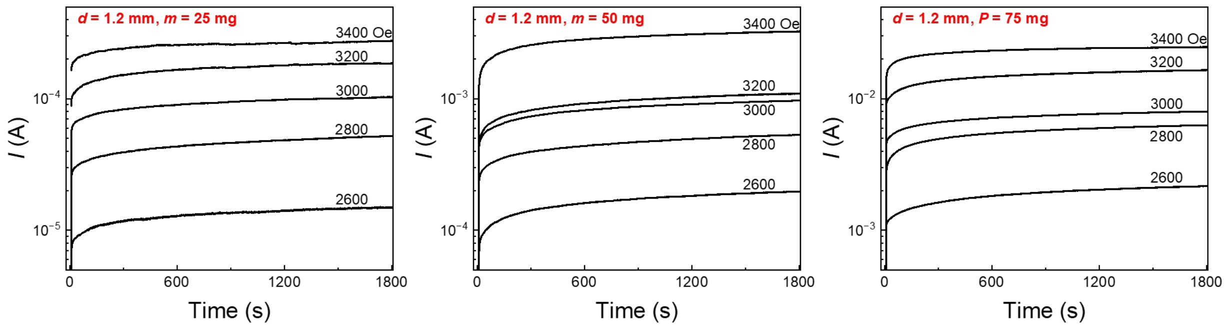

Figure S6. *P*-dependent electric current of ECFCCs under different magnetic field. The distance between two electrodes is d = 1.2 mm, the area of the ECFCCs is 15 × 15 mm.

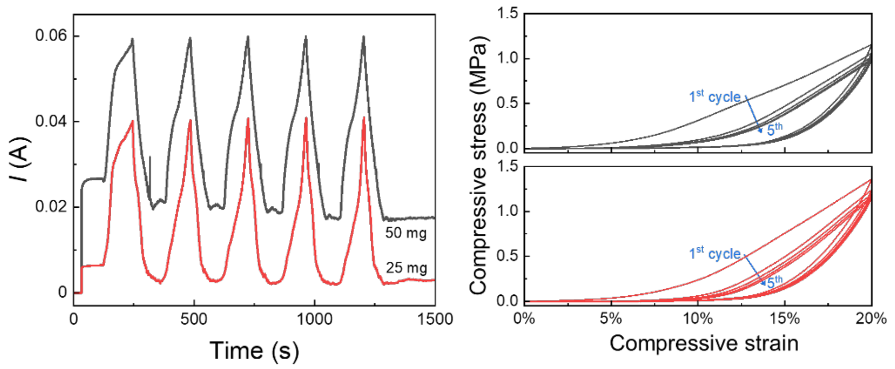

Figure S7. Repeatability of the resulting current under cycled compressions with an Instron machine. Default settings are 10 % / min pressing, 10 % / min releasing, max = 20 % and d = 1.5 mm (the distance between two glass slides). The findings demonstrate that the electrical responses of the setup are repeatable when subjected to consecutive in-situ measurements. The area of the ECFCCs is 15 × 15 mm.

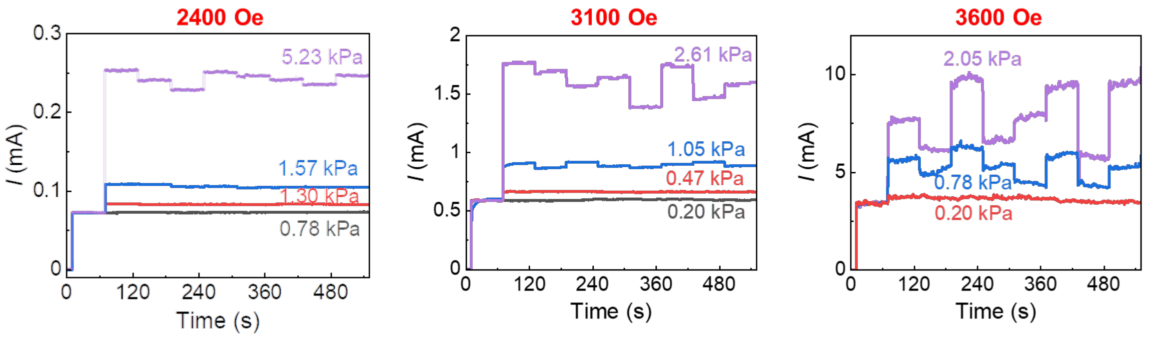

Figure S8. Representative electrical current responses upon the pressure responses under different H gatings. During the pressure sensing process, various H gatings were employed, and the device exhibited different response sensitivities to pressure stimuli, manifesting four distinct current response patterns. The first one is characterized by a relatively low sensitivity of the device to pressure stimuli under the corresponding H exposure, resulting in small changes in I when lighter objects exert pressure (black line). The second one occurs when H exposure enables detection of changes in the pillar structure induced by the pressure exerted by the object. However, due to a lower response sensitivity, it is unable to detect variations in the position of object (red line). The third one arises when H gating induces significant increases in I due to substantial changes in the pillar structure caused by the exerted pressure.

The device exhibits sufficient response sensitivity to accurately detect variations in the position of the weighed object (blue line). The last one is characterized by substantial increases in *I*, resulting from pronounced changes in the pillar structure caused by the exerted pressure. However, the device exhibits an excessively high response sensitivity, leading to significant variations in *I* depending on the placement of the object, which limits the accuracy of the final results (purple line), probably due to jamming effects.

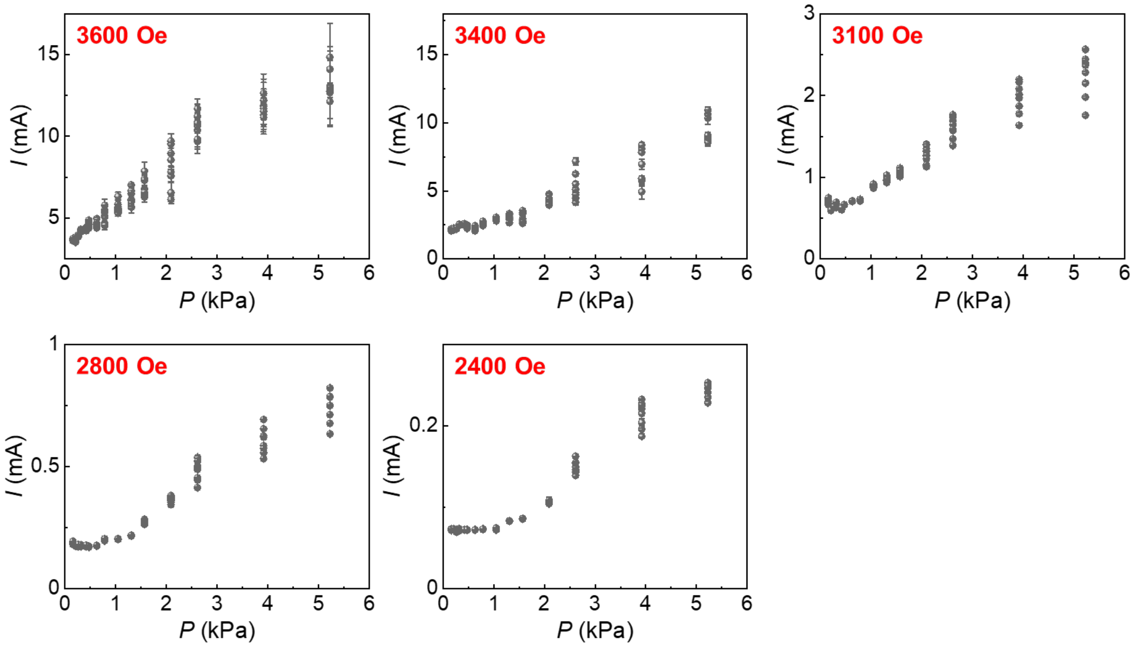

Figure S9. The results for five distinct *H* gating conditions, where each data point represents the average current corresponding to the external pressure during the sensing process. For each pressure level, eight data points were collected, representing measurements taken at eight different locations on the setup.

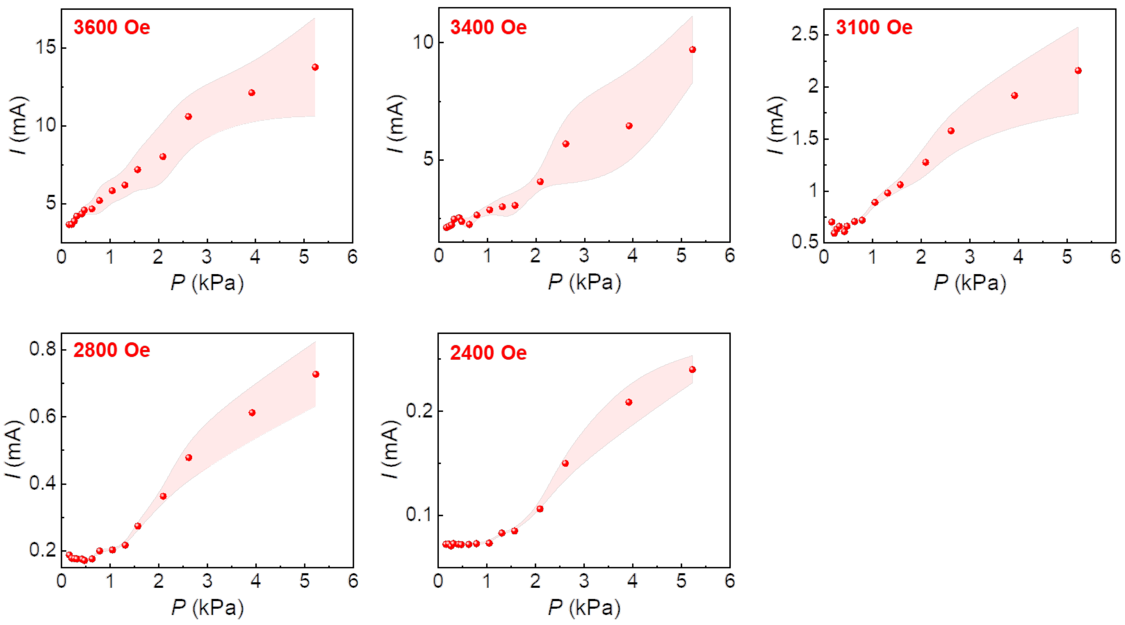

Figure S10. The average, maximum, and minimum values of the eight data points for each pressure in (Figure S8) are depicted by the dots and the borders of error bands, respectively.

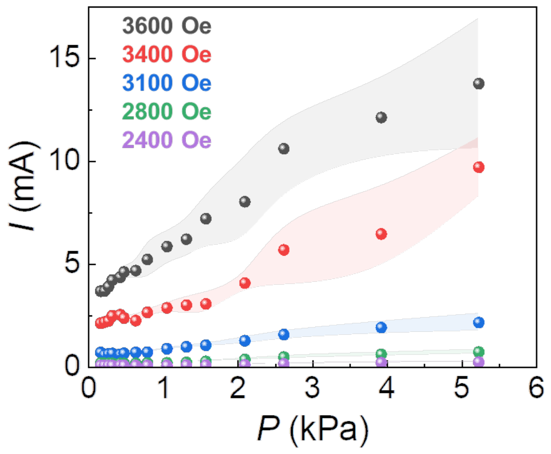

Figure S11. The correlation between external pressure and current during the sensing process under various magnetic field gatings.

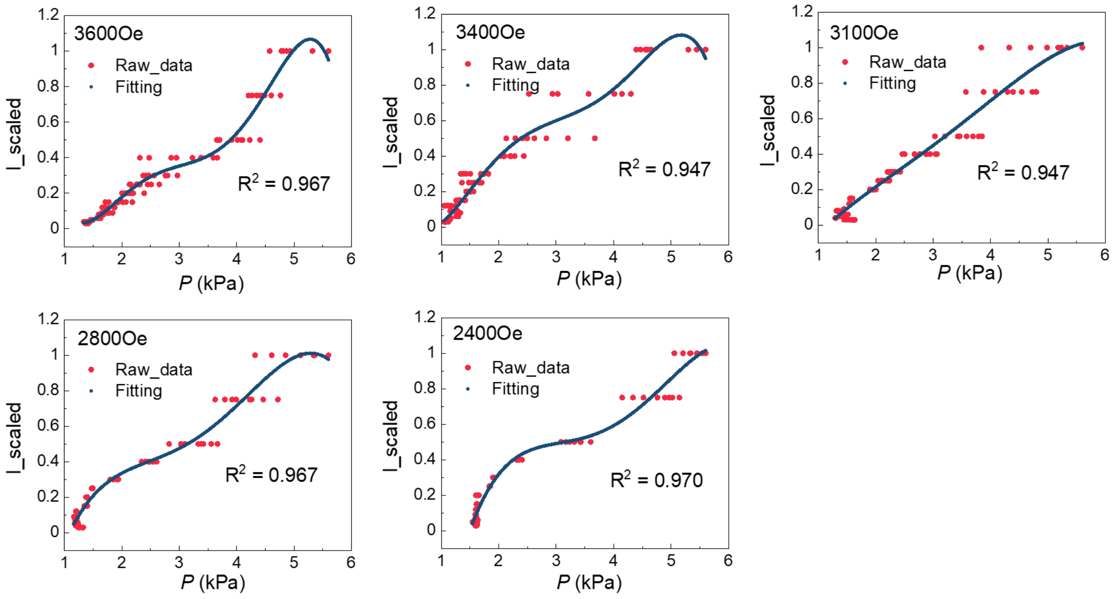

Figure S12. Polynomial fitting and evaluation on the current - pressure correlations at different field *H* gatings.

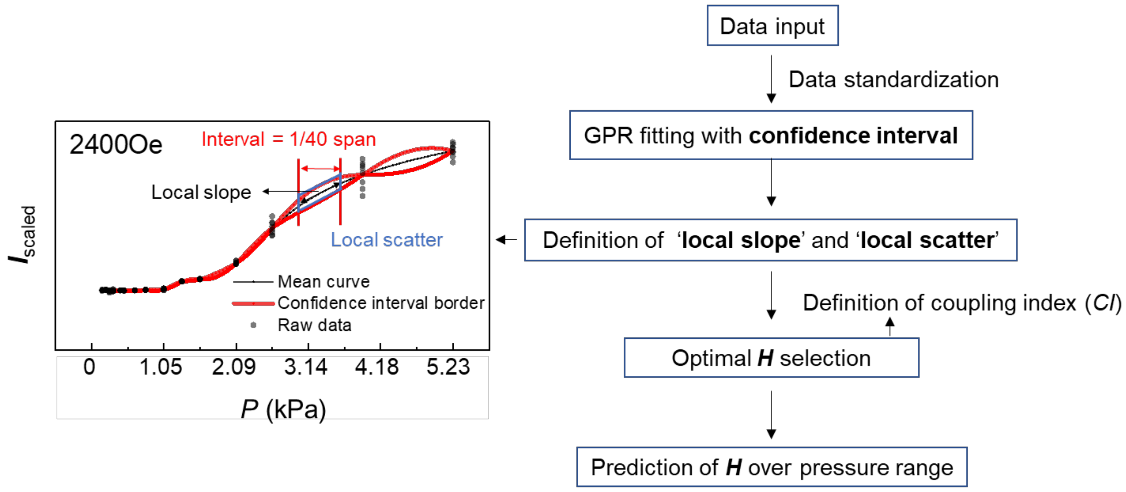

Figure S13. The workflow to establish optimal *H* selection. First, collected data were processed using MaxAbsScaler (In the scikit-learn Python package) prepared for GPR fitting. Then, the definitions of 'local slope' and 'local scatter' were proposed that, within the interval (1/40 span of the pressure range), local slope is calculated as the slope of the mean value (black curve) and local scatter is the calculated area within the interval. In the end, a coupling index (*CI*) (Equation S1) is defined as the quantitative measure for *H* selection, that the field scores the highest in *CI* value will be chosen as the optimal *H*.

$$Coupling\ index\ (CI) = \frac{local\ slope}{local\ scatter} \quad (S1)$$

Here in **Equation S1**, the coupling index (*CI*) is defined as the ratio of local slope to local scatter. The magnetic field with the highest *CI* value is identified as the optimal under given conditions.

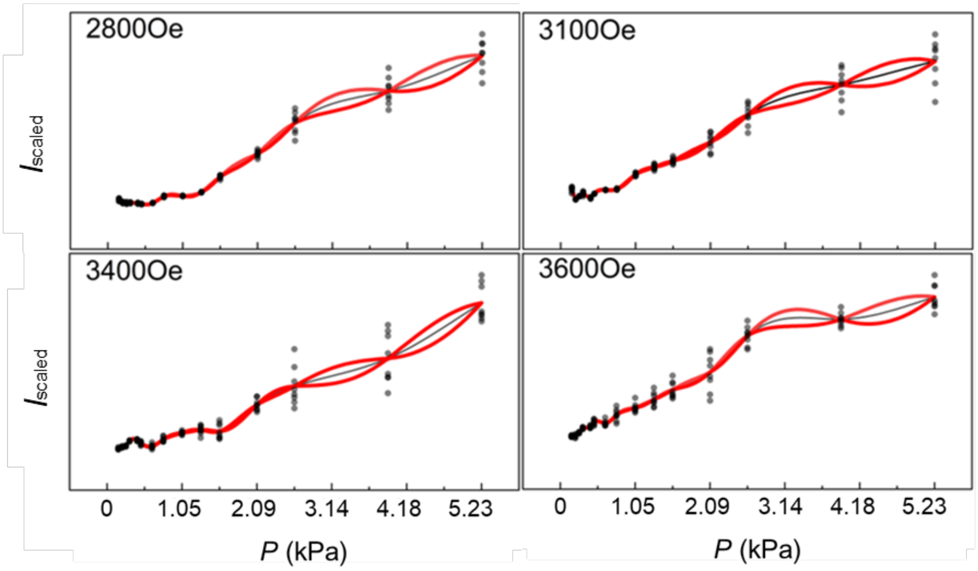

Figure S14. GPR fitting for different *H* gatings.

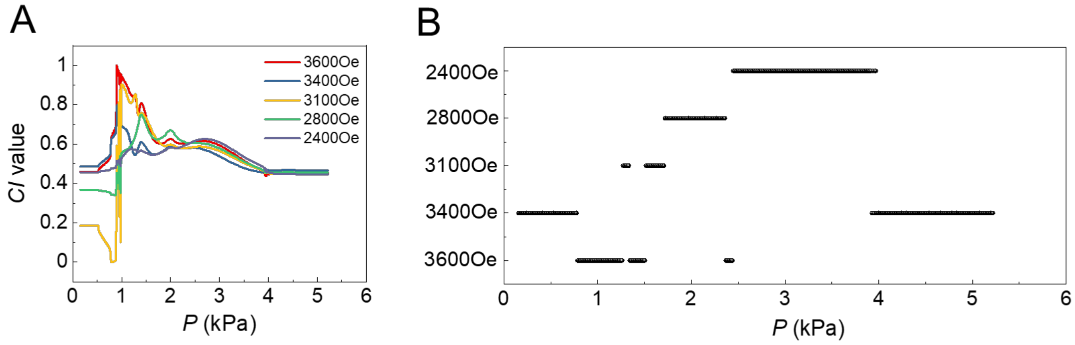

Figure S15 (A) Distribution of the calculated *CI* value at five different *H* over the pressure range. (B) Prediction of the optimal *H* corresponding to the highest *CI* value over the pressure range. The variation trend of the optimal *H* indicates the preferred smaller *H* for high pressures, and conversely, higher *H* for low pressures. This map captures the training data, suggesting the ideal *H* mode for different pressure range.

While the current results demonstrate the model's capability, they are not yet fully optimized due to the limited resolution of H and the restricted pressure range. To further refine the ML model, increasing the resolution (e.g., using a continuous range of H values) and expanding the overall pressure range would be beneficial. However, this would require significantly more data, as the broader parameter space introduces added complexity, which is currently beyond the scope of this study.

Table S1. Model evaluation results that confirm the model reliability. Cross validation verifies the generalization performance of the built model with the fold set at 5.

	MSE	RMSE	MAE	Score (R^2)	Cross validation
					MSE
3600 Oe	0.02	0.14	0.09	0.98	0.03
3400 Oe	0.12	0.33	0.17	0.93	0.07
3100 Oe	0.11	0.33	0.15	0.90	0.05
2800 Oe	0.02	0.14	0.07	0.98	0.02
2400 Oe	0.03	0.18	0.08	0.97	0.01

Note S1. Code for pressure prediction

```
import pandas as pd
import matplotlib.pyplot as plt
from sklearn.gaussian_process import GaussianProcessRegressor
from sklearn.gaussian_process.kernels import ConstantKernel, DotProduct, Matern
from sklearn.preprocessing import MaxAbsScaler, PolynomialFeatures
from sklearn.linear_model import LinearRegression
from sklearn.pipeline import make_pipeline
from scipy.integrate import trapz


raw_data = pd.read_csv(' ')
x = raw_data['X'].to_numpy().reshape(-1, 1)
scaler_x = MaxAbsScaler().fit(x)
X_scaled = scaler_x.transform(x)

y_columns = ['y1', 'y2', 'y3', 'y4', 'y5']
y_data_scaled = [ ]
scalers = [ ]
for col in y_columns:
    y = raw_data[col].to_numpy().reshape(-1, 1)
    scaler = MaxAbsScaler().fit(y)
    scalers.append(scaler)
    y_data_scaled.append(scaler.transform(y))

# GPR model definition
kernel = 1.2 * ConstantKernel(1.0) * DotProduct() + 1.3 * Matern(length_scale=3.0)
gpr = GaussianProcessRegressor(kernel=kernel, random_state=0)

# definition of local area and slope
def calculate_area_slope(x1, x2, model):
    x_lin = np.linspace(x1, x2, 100).reshape(-1, 1)
    y_mean, sigma = model.predict(x_lin, return_std=True)
    lower_bound = y_mean - 1.96 * sigma
    upper_bound = y_mean + 1.96 * sigma
    area = trapz(upper_bound - lower_bound, x=x_lin.ravel())
    slope = (model.predict(x2.reshape(-1, 1)) - model.predict(x1.reshape(-1, 1))) / (x2 - x1)
    return area, slope

# pressure estimation
def estimate_pres(y_data, X_scaled, input_I):
    poly_model = make_pipeline(PolynomialFeatures(4), LinearRegression())
    poly_model.fit(y_data, X_scaled)
    return poly_model.predict(input_I)

# optimal field selection
def field_selection(input_I, index, X_scaled, gpr, y_data_scaled):
    local_areas = []
    local_slopes = []
    coupling_vars = []
    coef = 2.5
    scat = coef * (max(X_scaled) - min(X_scaled)) / 100
    pres = estimate_pres(y_data_scaled[index], X_scaled, input_I)

    for y_scaled in y_data_scaled:
        model = gpr.fit(X_scaled, y_scaled.ravel())
```

```
        area, slope = calculate_area_slope(pres - scat, pres + scat, model)
        local_areas.append(area)
        local_slopes.append(slope)

    coupling_var = [a/b for a, b in zip(local_slopes, local_areas)]
    coupling_vars.append(coupling_var)
    np.argmax(coupling_var)
    return np.argmax(coupling_var)

test_data = { }
df_data = pd.DataFrame.from_dict(test_data, orient='index')

opt_Bs_dict = {}
for index, field in enumerate(df_data.index):
    opt_Bs = []
    for value in df_data.loc[field]:
        val_scaled = scalers[index].transform(np.array(value).reshape(-1, 1))
        opt_B = field_selection(val_scaled, index, X_scaled, gpr, y_data_scaled)
        opt_Bs.append(opt_B)
    opt_Bs_dict[field] = opt_Bs

for field, opt_Bs in opt_Bs_dict.items():
    print(f"Default field: {field}, optimal fields: {opt_Bs} ")
```

References

(1) Liu, X. H.; Tan, H. W.; Rigoni, C.; Hartikainen, T.; Asghar, N.; Dijken, S. V; Timonen, J. V. I.; Peng, B.; Ikkala, O. Magnetic field-driven particle assembly and jamming for bistable memory and response plasticity. *Sci. Adv*. **2022**, *8*, eadc9394.

(2) Peng, B.; Zhang, X. L.; Aarts, D. G. A. L.; Dullens, R. P. A. Superparamagnetic nickel colloidal nanocrystal clusters with antibacterial activity and bacteria binding ability. *Nat. Nanotechnol*. **2018**, *13*, 478–482.

(3) Liu, X. H.; Tan, H. W.; Stråka, E.; Hu, X. H.; Chen, M.; Sebastiaan, v. D.; Scacchi, A.; Sammalkorpi, M.; Ikkala, O.; Peng, B. Trainable bioinspired magnetic sensitivity adaptation using ferromagnetic colloidal assemblies. *Cell. Rep. Phys. Sci*. **2024**, *5*, 101923.

(4) Schulz, E.; Speekenbrink, M.; Krause, A.; A tutorial on Gaussian process regression: Modelling, exploring, and exploiting functions. *J. Math. Psychol*. **2018**, *85*, 1–16.

(5) Lazaro-Gredilla, M.; Quinonero-Candela, J.; Rasmussen, C. E.; Figueiras-Vidal, A. R. Sparse Spectrum Gaussian Process Regression. *J. Mach. Learn. Res*. **2010**, *11*, 1865–1881.
